# The Physician’s Visiting List, Diary and Book of Engagements for 1852

**Published:** 1852-01

**Authors:** 


### ARTICLE XIII.

*The Physician's Visiting List, Diary and Book of Engagements for* 1852. Philadelphia: Lindsay & Blakiston. (From the Publishers through S. C. Griggs & Co.)

This will be especially acceptable to the systematic practitioner as it will readily enable him to keep everything pertaining to the business part of his practice in good order.

It contains an almanac for the year, a table of doses proportioned to the different ages of patients, a list of poisons with their antidotes, an extract from the code of ethics of the American Medical Association defining the duties of Physicians to each other and the profession at large, blank leaves with printed heads for visiting list, general memoranda, obstetric and vaccination engagements, list of things lent, &c., &c.

It is of convenient size to carry in the pocket, and is got up in excellent style.

